# Electric Blue: Molecular Evolution of Three-Finger Toxins in the Long-Glanded Coral Snake Species *Calliophis bivirgatus*

**DOI:** 10.3390/toxins13020124

**Published:** 2021-02-08

**Authors:** Daniel Dashevsky, Darin Rokyta, Nathaniel Frank, Amanda Nouwens, Bryan G. Fry

**Affiliations:** 1 Venom Evolution Lab, School of Biological Sciences, University of Queensland, St Lucia, QLD 4072, Australia; danieldashevsky@gmail.com; 2 Australian National Insect Collection, Commonwealth Science and Industry Research Organization, Canberra, ACT 2601, Australia; 3 Department of Biological Sciences, Florida State University, Tallahassee, FL 24105, USA; drokyta@bio.fsu.edu; 4 MToxins Venom Lab, 717 Oregon Street, Oshkosh, WI 54902, USA; nate@mtoxins.com; 5 School of Chemistry and Molecular Biosciences, University of Queensland, St Lucia, QLD 4072, Australia; a.nouwens@uq.edu.au

**Keywords:** blue coral snake, three-finger toxin, transcriptomics, venomics

## Abstract

The genus *Calliophis* is the most basal branch of the family Elapidae and several species in it have developed highly elongated venom glands. Recent research has shown that *C. bivirgatus* has evolved a seemingly unique toxin (calliotoxin) that produces spastic paralysis in their prey by acting on the voltage-gated sodium (Na_V_) channels. We assembled a transcriptome from *C. bivirgatus* to investigate the molecular characteristics of these toxins and the venom as a whole. We find strong confirmation that this genus produces the classic elapid eight-cysteine three-finger toxins, that δ-elapitoxins (toxins that resemble calliotoxin) are responsible for a substantial portion of the venom composition, and that these toxins form a distinct clade within a larger, more diverse clade of *C. bivirgatus* three-finger toxins. This broader clade of *C. bivirgatus* toxins also contains the previously named maticotoxins and is somewhat closely related to cytotoxins from other elapids. However, the toxins from this clade that have been characterized are not themselves cytotoxic. No other toxins show clear relationships to toxins of known function from other species.

## 1. Introduction

In many respects, snakes of the family Elapidae are among the most notable worldwide. Members of the family, such as cobras (genus *Naja*) and the black mamba (*Dendroaspis polylepis*), are famous beyond the confines of herpetology and command instant recognition and respect across cultures and languages. This is due in large part to the potent venoms that these snakes possess. Elapids are the most speciose of the three lineages of front-fanged snakes and, along with the family Viperidae, comprise the vast majority of medically significant snake species [1,2,3]. Elapids are a prominent component of human-snake conflict in much of Sub-Saharan Africa, across Asia, and through Papua New Guinea and Australia [4,5,6]. Of the ‘Big Four’ species of medically important snakes in India, two are elapids: *Naja naja* (the Indian cobra) and *Bungarus caeruleus* (the common krait) [7]. The latter poses a non-standard challenge to snakebite prevention because many of the bites from this species are a result of the snake entering human houses at night while the occupants are sleeping [8]. Similar behavior has also been reported in some African species of the genus *Naja* [9,10].

The origins of the elapids remain somewhat unclear. They likely arose in Africa or Asia around 30 million years ago before quickly radiating out to southern Asia, Australia, and the Americas, as well as the Pacific and Indian oceans [11,12,13]. What triggered this radiation remains uncertain. The most obvious candidate feature that may have contributed to their success is their venom system. Elapids are one of three families of front-fanged snakes along with viperids and atractaspids [14]. In non-front-fanged snakes, ducts leading from the venom glands open into the mouth near the back of the maxilla; many species have adaptations to enhance the delivery of venom, such as enlarged or grooved teeth associated with the duct opening [15]. All three of the front-fanged lineages have independently evolved maxillae that are reduced in size and number of teeth, which brings these anterior teeth and associated venom ducts towards the front of the mouth which makes envenomation a mechanically simpler process [16]. At the same time, these families’ teeth were further adapted to become large (especially in Viperidae and Atractaspidae) tubular fangs which can conduct venom directly from the venom duct into the snake’s target [17]. Along with these changes in dentition, front-fanged snakes developed compressor muscles to eject venom from the lumen of the venom gland, along the venom duct, and out the end of the fang [18]. The elapid venom delivery system is distinct from the other front-fanged families in that slightly different muscles were co-opted for use as a venom gland compressor muscle, and, unlike the large mobile fangs of viperids and atractaspids, their fangs are relatively short and fixed in place relative to the rest of the skull, which are better suited to the hard scales of reptilian prey preferred by many elapids [19,20].

Beyond even the morphological adaptations, the most important part of a venom system is the venom itself. Most elapid venoms are neurotoxic, and the most prevalent neurotoxins across the family belong to the three-finger toxin (3FTx) protein family [18,21]. These toxins are estimated to have first appeared over 100 million years ago, relatively early in the evolutionary history of snakes, and, as a result, have been reported from transcripts of most families of alethinophidian snakes [22,23]. The toxin family derives its name from the characteristic tertiary structure of three β-stranded loops, or ‘fingers’, that emerge from a core that is stabilized by cysteine-cysteine bonds [24]. The ancestral forms of 3FTx contain ten cysteine residues which form five disulfide bonds between particular pairs [21,25]. 3FTx are most prevalent in the venoms from the families Colubridae and Elapidae [26]. While colubrid snakes retain the plesiotypic ten-cysteine 3FTx, a common ancestor of elapids evolved a form of 3FTx that did not include the second and third ancestral cysteines, leaving only four pairs; these apotypic 3FTx diversified greatly and are far more highly expressed in elapid venoms than the ten-cysteine forms [27,28].

All the ten-cysteine 3FTx that have been characterized show similar toxic activities: binding to and thereby blocking the activity of nicotinic acetylcholine receptors on the skeletal muscles [21,29,30]. This results in flaccid paralysis and is often referred to as α-neurotoxicity and these toxins usually display a greater affinity for the receptors of diapsids (birds and reptiles) than those of synapsids (mammals) [31,32]. Even novel modifications to the structure of these toxins, such as the covalently bonded heterodimers from the colubrid species *Boiga irregularis* and its close relatives, increase the toxicity rather than changing the activity of these toxins [33,34]. The derived eight-cysteine toxins found in elapids, on the other hand, have evolved a wide array of functions beyond just the plesiotypic α-neurotoxicity, including κ-neurotoxicity (α-neurotoxicity guided by a non-disulfide linked dimer), presynaptic neurotoxicity by binding to calcium channels, and non-neurotoxic activities, such as cytotoxicity and platelet inhibition [27,28,29,35,36]. Many of the eight-cysteine toxins that did retain the ancestral activity have evolved greatly increased toxicity to the extent that α-bungarotoxin has seen widespread use as an experimental tool because of its potency and specificity [24,37]. These 3FTx are the primary functional toxins in many elapid venoms and are responsible for the danger posed by many species, including those from the genera *Naja* (cobras), *Dendroaspis* (mambas), *Micrurus* (coral snakes), and the subfamily Hydrophiinae (sea snakes and Australian elapids) [38,39,40,41,42].

Given that these unique and potent eight-cysteine 3FTx have been found in virtually all elapid venoms that have been studied to date, it is clear that they first evolved very early in the evolutionary history of the family, perhaps in the common ancestor of all extant elapids. However, it remains unclear if either the origin of these unique toxins or the morphological adaptations to more effectively deliver them preceded the other or whether they evolved in concert. One way to address questions about the order in which these key innovations evolved and what role they might have played in the radiation of the elapids is to examine the venom system of the most basal members of the family. The ecology, anatomy, and physiology of these snakes may provide insight about the niche occupied by the common ancestor of elapids and the state of these characteristic traits in those species may inform us about their evolutionary trajectories.

**Figure 1:**
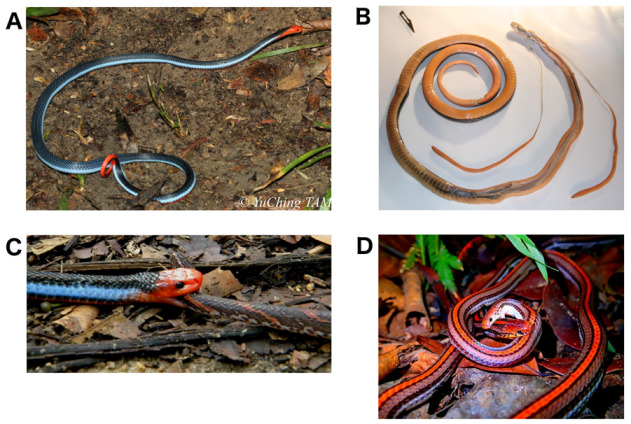
(**A**) The striking colors of *Calliophis bivirgatus*, image by Yu Ching Tam via iNaturalist under CC BY-NC; (**B**) the dissected venom glands of *C. bivirgatus*; (**C**) *C. bivirgatus* preying upon *Oligodon signatus*, image by Xu Weiting [49]; (**D**) attempted cannibalism by *C. intestinalis*, image by Gan Gim Chuah via iNaturalist under CC BY-NC.

Modern phylogenies virtually all agree that the genus *Calliophis* (Asian coral snakes) is the most basal clade in the elapid radiation [43,44,45]. Most of the fifteen described species species across their South and Southeast Asian distribution are small, semi-fossorial, and feed on elongate prey (Figure 1) [3,46,47,48,49]. However, within the genus, there is a particular subclade that stands out in terms of external and internal morphology [11,45,50]. Referred to as long-glanded *Calliophis*, these 7 species (*C. bilineata*, *C. bivirgatus*, *C. intestinalis*, *C. phillipina*, *C. salitan*, and *C. suluensis*) are large-bodied with high-visibility patterns even on the dorsal side (many of the short-glanded species also use aposematic underbellies), primarily ophiophagous, and possess unusually enlarged venom glands that extend from the back of the head (where most elapid venom glands are located) into the body cavity and can reach up to 1/3 of the body length (Figure 1B) [3,47,48,51,52,53]. The exact relationships between short and long-glanded *Calliophis* remain somewhat unclear [45]. Despite their broad geographical range, there are few reports of humans being bitten by species of this genus. The most recent of these reports briefly discussed the entire available literature on the matter [54]. From this overview the conclusion was that there is no evidence of severe envenomation from short-glanded species [54,55,56,57], *C. intestinalis* have proven more dangerous but non-fatal [58,59,60], and the only death from the genus is attributed to *C. bivirgatus* [61,62,63].

Several lines of in vitro evidence also underscore the danger of bites from *C. bivirgatus*. There is no specific antivenom developed against *Calliophis* venom, and preliminary tests of local elapid antivenoms found that the most effective antivenom still required very large doses to protect mice from challenges of 2.5 LD${}_{50}$ and was ineffective against challenges of 5 LD${}_{50}$ [64]. A subsequent study that involved a wider range of regional antivenoms noted that “[a]gainst a lethal challenge dose (2.5 LD50) of *C. bivirgat*[*us*] venom, all tested antivenoms failed to protect the mice, with the animals noted to die in spasticity (convulsion-like feature with myoclonus followed by muscle spasm, curled front limbs and out-stretched hind limbs) within 1 min post-injection of the venom-antivenom mixtures” [65]. These symptoms could be explained by experiments that demonstrate that *C. bivirgatus* venom contains a toxin (known as calliotoxin) which functionally act as agonists to voltage-gated sodium channels associated with skeletal muscle (Na_V_1.4) by delaying the inactivation of these channels; when added to an organ bath, the venom elicited spontaneous contractions from nerve-muscle preparations [52]. These spasms develop rapidly and may help explain the relatively swift death of the one confirmed bite victim of *C. bivirgatus* [61,62]. The effects of these toxins are functionally equivalent to the spastic paralysis that electric eels produce in their prey by the application of high-voltage electric discharge [66,67].

This toxic activity is in stark contrast to the usual α-neurotoxicity of elapid snakes. The clade referred to as the true coral snakes (containing the genera *Sinomicrurus*, *Micruroides*, and *Micrurus*) are a good point of comparison to *Calliophis* evolutionarily and ecologically. This clade forms the next most basal branch of the elapid phylogeny after *Calliophis* [13,43,44,68] and most of the species that belong to it are fossorial, brightly-colored, and specialize in eating elongate ectothermic prey [69,70,71]. The symptoms that occur when these snakes bite humans are markedly different from those produced by bites from long-glanded *Calliophis*. For instance, the single published account of a *Sinomicrurus macclellandi* bite indicated that the victim felt nothing until motor issues started developing 6 h after the bite and culminated in lethal respiratory paralysis after 8 h [72]. Similarly, bites from snakes of the genus *Micrurus* often exhibit few symptoms besides paralysis [73,74,75].

Along with studies of the overall effect of *C. bivirgatus* venom, there have also been several lines of research into the molecular composition thereof. Takasaki et al. [76] isolated a number of toxins, tested their activities, and generated partial sequences, including maticotoxin A, which they noted bears some resemblance to cytotoxins from other elapids. Tan et al. [64] studied the proteomics of the venom and again noted the similarity of some of the toxins to known cytotoxins but, due to the general lack of published *Calliophis* sequences to include in their reference database, were not able to reach specific conclusions about these toxin sequences. This study did, however, demonstrate mild cytotoxic activity from the venom which is consistent with the slight local effects of the one detailed bite report [61,62]. Yang et al. [52] identified calliotoxin as the toxin responsible for the unusual spastic paralysis the venom causes, generated a partial amino acid sequence, and matched it to a sequence from a preliminary assembly of the transcriptome that we analyze in this paper. Taken together, the evidence was strong that *Calliophis bivirgatus* possesses the apotypic elapid 8-cysteine 3FTx. This is not necessarily a surprising finding, but, due to the evolutionary position of *Calliophis*, it does have implications that were not explored in these previous studies. Our aim with the full transcriptome is to reinforce this finding, examine the full diversity of the 3FTx present in the transcriptome, and discuss what these results might suggest about the evolutionary history of 3FTx in *Calliophis* and Elapidae as a whole.

## 2. Results & Discussion

**Figure 2:**
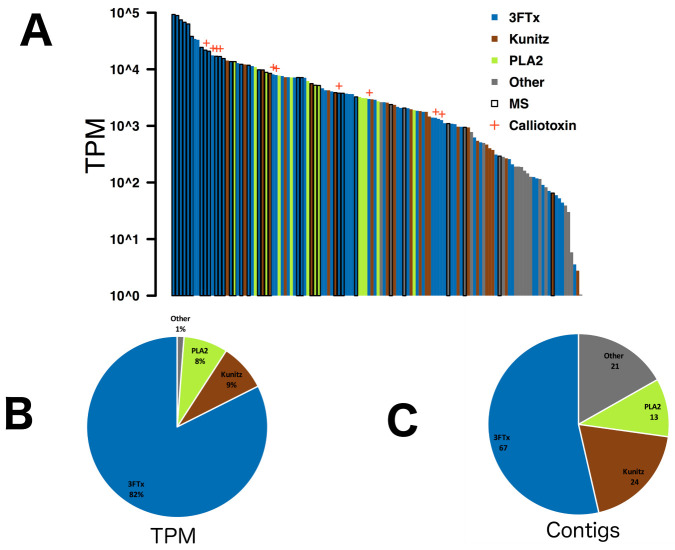
The venom gland transcriptome of *Calliophis bivirgatus* is dominated by three-finger toxin (3FTx), kunitz peptides, and phospholipase A2 (PLA_2_): (**A**) Transcripts Per Million (TPM) normalized abundance levels for all 125 toxin contigs, black borders to indicate those which had matches in our proteomics, orange crosses indicate δ-elapitoxins; (**B**) proportion of total TPM accounted for by the major toxin families; (**C**) total number of unique contigs recovered from the major toxin families.

Our *Calliophis bivirgatus* transcriptome contained 125 unique toxins after rigorous quality control and filtering. After expression levels were normalized to represent the relative abundance of the transcripts using the formula for Transcripts Per Million (TPM), only three toxin families had a combined expression level greater than 1% of the total. In descending order of both expression level and number of unique contigs these families were 3FTx, kunitz peptides, and phospholipase A2s (PLA_2_s; Figure 2). The remaining 21 toxin contigs include cysteine-rich secretory proteins (CRiSPs), cystatins, hyaluronidase, kallikrein, nerve growth factors, natriuretic peptides, phopsphodiesterase, phospholipase B, snake venom metalloproteases (SVMPs), vespryn, and waprin; SVMPs are common in many other snake venoms (espcially viperid or colubrid venoms), while the other families have been reported from a variety of venom transcriptomes, but are rarely if ever recovered in notable quantities from actual venom and their functions remain almost entirely unknown [77]. Many of these contigs matched to the spectra of ion fragments produced by MS/MS proteomics, including 15 of the 20 highest expressed toxins, which included 3FTx, PLA_2_s, kunitz peptides, SVMPs, CRiSP, and vespryn (Full MS/MS results can be found in the Supplementary Materials). These results offer robust confirmation that eight-cysteine 3FTx are present in the most basal elapids, which implies that the common ancestor of Elapidae seems to have already possessed eight-cysteine 3FTx, fixed front fangs, and gland compressor muscles. Whether these traits evolved sequentially or coevolved more or less simultaneously, this suggests that the complete venom system may have been a crucial factor in the explosive radiation of the family.

**Figure 3:**
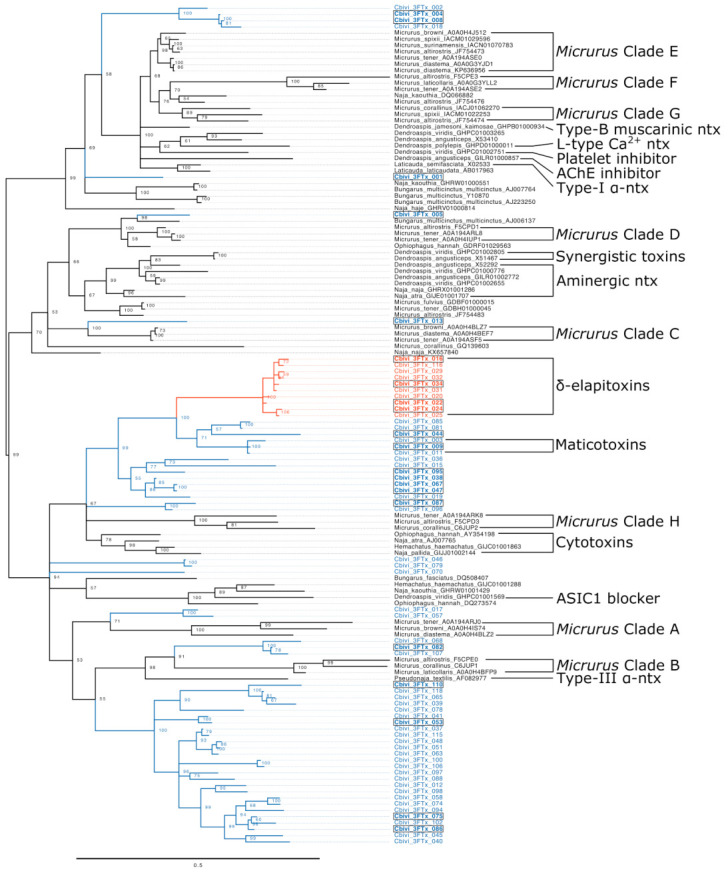
Unrooted tree of representative eight-cysteine 3FTx from across major taxonomic and functional divisions (black), as well as those from *Calliophs bivirgatus*. Clades of *Micrurus* toxins are labeled in accordance with Dashevsky and Fry [78]. Most *C. bivirgatus* toxins are colored blue, while δ-elapitoxins are colored orange. *C. bivirgatus* toxins which had matches in our proteomics are bolded and boxed. Scale bar represents an average of 0.5 substitutions per site, and node labels indicate % support values.

Given that *Calliophis* is the most basal extant genus that produces the eight-cysteine 3FTx characteristic to the elapids, we hoped that the sequences in this transcriptome would help clarify the evolution of these toxins. Our phylogenetic analysis puts the 3FTx recovered from *C. bivirgatus* into the context of the previously known diversity of the toxin family (Figure 3). The small size and high rates of evolution of the 3FTx lead to extreme diversity and mutation saturation at many sites which make investigations of their precise history difficult, so many analyses of these toxins have resorted to creating unrooted phylogenies [23,34,78]. Because of this, it is currently unclear which clades of eight-cysteine 3FTx sit near the base of the radiation after they evolved from the 10-cysteine forms. Our analyses suffer from these same problems, and we also make use of an unrooted tree (in Figure 3, a midpoint root was chosen for the purposes of visual presentation) but offer some suggestive results regarding the evolutionary history of the structure and activity of this toxin family. The fact that *C. bivirgatus* toxins can be found across the tree suggests that the eight-cysteine 3FTx had already begun to duplicate and diversify before the *Calliophis* separated from the other elapids.

**Figure 4:**
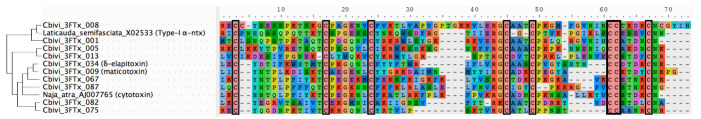
Amino acid alignment of representative sequences from the various clades of *C. bivirgatus* 3FTx (cladogram on the left is derived from the topology of the more comprehensive phylogeny in Figure 3). Sequences include representatives from each clade where at least one member was confirmed via MS/MS, both the δ-elapitoxins and the maticotoxins, as well as a cytotoxin from *Naja atra*, for comparison to those closely related toxins and a type-I α-neurotoxin from *Laticauda semifasciata* as the ‘stereotypical’ sort of 8-cysteine 3FTx. The eight conserved cysteine residues are highlighted with boxes.

The majority of *C. bivirgatus* 3FTx, including the δ-elapitoxins, are contained within two monophyletic clades that do not include toxins from any other species. Based on previous research, we have annotated two subclades within one of these as the δ-elapitoxins and the maticotoxins. The δ-elapitoxins are a distinct clade that is relatively divergent from its nearest relatives but similar within the group (a long branch leads to the clade, but the internal branches are short); these sequences closely resemble the published sequence of calliotoxin [52] (See Figure 4). While Takasaki et al. [76] named several maticotoxins, they only published a partial sequence from maticotoxin A, so, out of conservatism, we have only annotated a small clade of sequences that resemble that fragment as maticotoxins (See Figure 4). Since only gross amino acid compositions were published for the other three maticotoxins referred to by Takasaki et al. [76], we may never be able to associate them with specific sequences. No *C. bivirgatus* 3FTx can easily be associated with toxins of known function from other species. While previous studies have repeatedly noted the sequence similarity between certain *C. bivirgatus* toxins and the cytotoxins expressed in more derived elapids [64,76], we find this comparison to be unhelpful. While it is true that the cytotoxins are relatively close neighbors of the clades that contain the δ-elapitoxins and the maticotoxins, the studies that described both of those noted that they themselves are not cytotoxic [52,76]. Moreover, the clade that contains those two groups is part of a polytomy that includes another pair of *C. bivirgatus* toxins, representatives of a large clade of *Micrurus* toxins which have never been shown to be cytotoxic, and the cytotoxins themselves. This suggests that many if not all of the derived functions that have been discovered in the 3FTx of other elapids are evolutionary innovations that postdate the split between *Calliophis* and the rest of the family.

We analyzed signals of natural selection in the coding sequence of both large clades of *C. bivirgatus* 3FTx and found that they are subject to strong positive selection. The ratios of non-synonymous and synonymous mutations ($\frac{dN}{dS}$) was estimated to be 2.40 for the clade containing δ-elapitoxins and the maticotoxins and 2.37 for the other clade. These strong signals of positive selection are often found in analyses of 3FTx [34,35,78,79] and are the likely the cause of the rapid rates of evolution that the toxin family exhibits. Positive selection can be the result of biological arms races, which are a relatively common scenario in toxin evolution. Indeed, a predator-prey arms race has likely played a role in the evolutionary history of *C. bivirgatus* 3FTx. Recent studies have shown that different modes of resistance to α-neurotoxins are widespread in snakes, including charge reversal lysine mutations that have arisen many times in snakes [80] and an *N*-glycosylation mutation that grants near-total immunity to the standard α-neurotoxic 3FTx [81,82]. For any ophiophagous snake, and especially one that routinely preys upon elapids, like the long-glanded *Calliophis* do (Figure 1D) [3,46,47,48,49], any way around this resistance in potential prey items would likely be beneficial as it would greatly increase the amount of prey the snake could reasonably feed on. The evolution of δ-elapitoxins that target an entirely different family of receptor would have neatly sidestepped the issue of α-neurotoxin resistance.

These arms races could also be relevant to the question of why a lineage of *Calliophis* evolved such extraordinarily long venom glands. They are likely certainly a compromise between the need to produce larger venom yields while maintaining a slim profile. *Calliophis* are all semi-fossorial, and their slender build allows them to move through leaf litter, soil, and undergrowth with relatively little resistance. This lifestyle would be hindered by the development of large venom glands, like those found in some cobras or Australian elapids, which increase in circumference and give those snakes very broad heads. In the African viperid genus *Causus*, one species has developed similarly elongated glands, and this is hypothesized to be in response to niche partitioning driving that species to take larger prey items [83]. One possible explanation for this adaptation in *Calliophis* is that, prior to the evolution of Na_V_ toxins, the ancestral long-glanded lineage adapted to α-neurotoxin resistance in their prey with increased venom yield. However, that does not explain why the development of these elaborate structures has not been subsequently selected against now that these species possess toxins which circumvent that resistance. Additionally, there are several other lineages of elapids (e.g., *Bungarus* and *Micrurus*) that face similar selective pressures but have not developed enlarged venom glands of any sort and primarily employ neurotoxins that cause flaccid paralysis. In other snake species, some researchers have proposed that snakes may evolve overkill venoms for a number of reasons [84,85]; those same pressures could potentially account for the increased yield requirements leading to the large glands.

The possibility also presents itself that the elongated glands may have co-evolved with the Na_V_ channel toxins similar to how eight-cysteine 3FTx may have co-evolved with the front fangs and gland compressor muscles in the common ancestor of all elapids. However, such a hypothesis cannot be determined from *C. bivirgatus* alone. More studies will have to be undertaken studying the toxins of both long and short-glanded *Calliophis* to attempt to reconstruct the evolutionary history of the genus’ venom system.

## 3. Materials and Methods

### 3.1. Venom and Tissue Acquisition

All venom work was undertaken under University of Queensland Institutional Biosafety Committee Approval #IBC134BSBS2015. Malaysian *Calliophis bivirgatus* venom and tissue from a captive specimen were provided by commercial supplier MToxins Venom Lab, Oshkosh, WI, USA (University of Melbourne ethics approval #UM0706247). Venom was snap-frozen in liquid nitrogen and then lyophilized. Tissue was stored in liquid nitrogen until defrosted for RNA extraction. All venoms and tissues were imported under Australian Quarantine and Inspection Service permit 0001804439.

### 3.2. RNA Extraction

Venom gland tissue (20 mg) was homogenized using a rotor homogenizer and total ribonucleic acid (RNA) extracted using the standard TRIzol Plus methodology (Invitrogen, Waltham, MA USA). RNA quantity was assessed using a Nanodrop 2000, spectrophotometer (Thermo Scientific, Waltham, MA USA). mRNA is extracted and isolated using standard Dynabeads mRNA DIRECT Kit (Life Technologies Ambion, Waltham, MA USA). Dynabeads work by hybridising poly-thymine (polyT) sequences, covalently bound to the Dynabead surface, to the mRNA poly-aderine (polyA) tail. This selectively enriches the sample for mRNA during the centrifugation and washing stages, since other RNA forms (which do not have a polyA tail) are removed. This facilitates a higher concentration of mRNA yield for sequencing purposes. RNA quality was checked on an Agilent 2100 BioAnalyzer instrument using the Agilent RNA Nano 6000 kit, PN 5067-1511 (Santa Clara, CA, USA).

### 3.3. Sequencing

High throughput sequencing was conducted by the Institute for Molecular Biosciences Sequencing Facility (Institute of Molecular Bioscience, UQ, St Lucia, Australia). Libraries were prepared using TruSeq Stranded mRNA Library Preparation Kit (Illumina, San Diego, CA, USA), following the manufacturer’s instructions, with 1.0 μg RNA input, a fragmentation time of 2 min, and 15 cycles of amplification. Libraries were sequenced on an Illumina NextSeq 500 (San Diego, CA, USA), using a 2 × 150 bp High Output V2 kit.

The transcriptome described in this paper was one of several species that were multiplexed together as part of a single sequencing run. Throughout our workflow, GNU parallel 20170822 was used to run similar tasks in simultaneously, CD-HIT 4.8.1 was used to remove duplicate sequences, and Seqtk 1.2 was used to obtain subsets of sequence files [86,87,88,89]. In addition, as a result of multiplexing, we experienced the common problem of low, but meaningful, amounts of cross contamination between our multiplexed samples [90]. We attempted to mitigate this issue at several points in our methodology.

### 3.4. Assembly

Illumina reads that were likely to be cross contamination between multiplexed samples were removed from our read files by identifying 57 nucleotide k-mers in our focal read set that were present in another read set from the same lane at a 1000-fold or higher level. Reads with 25% or more of their sequence represented by such k-mers were filtered from the data set. This was accomplished using Jellyfish 2.2.6 [91] and K-mer Analysis Toolkit (KAT) 2.3.4 [92]. We then removed adaptors and low-quality bases from the reads and removed any reads shorter than 75 base pairs using Trim Galore version 0.4.3 [93]. We then used PEAR 0.9.10 [94] to combine pairs of reads whose ends overlapped into one, longer, merged read. We then carried out several independent de novo assemblies of these reads using the programs Extender version 1.04 [95], Trinity version 2.4.0 [96], and SOAPdenovo version 2.04 [97]. SOAPdenovo was run repeatedly with k-mer sizes of 31, 75, 97, and 127. The raw reads may be found in the NCBI Sequence Read Archive associated with BioProject PRJNA698476.

### 3.5. Annotation

The de novo assemblies were concatenated and TransDecoder 5.2.0 was used to predict open reading frames (ORFs) within the contigs [98]. We removed any ORFs that did not include both a start and stop codon. The remaining ORFs were then compared against reference toxin sequences obtained from UniProt using standalone BLAST 2.6.0 [99,100,101]. The resulting ORFs with similarity to known toxins were further quality controlled by manual inspection and BLAST searches against the Reptilia subsection of the NCBI Nucleotide database [99,100,102]. The Burrows-Wheeler Aligner (BWA) 0.7.16a and SAMtools 1.5 were used to align the original reads from each species to the total list of annotated ORFs from every assembly [103,104]. These results were visualized using Tablet 1.15 to screen for signs of chimerical assembly [105]. Those that showed sharp discontinuities in coverage maps across all species were removed from further analysis. A combination of read coverage, assembly quality, and BLAST search results were used to confirm the species of origin for each ORF. Since the species that were multiplexed together all come from different families and the high rate of evolution in toxin genes, it is extremely unlikely that any two of these species would express identical toxins. Because of this, we interpret reads from multiple species aligning to a single contig as instances of contamination and tentatively assign the contig to the species with significantly higher expression levels. This assignation can be further reinforced if our BLAST results indicate that the sequence is question is more similar to the high-expression taxon than the lower one. The distribution of reads mapped to the sequence can also be informative if the pattern matches the norm for that toxin family in one species but not the others. For most sequences, all three indicators were unambiguous and in agreement while in a small minority of cases two other indicators could be used to decide between somewhat equivocal results of a third. No sequences that had made it to this point of the quality control process could not be confidently assigned using these methods. Once each ORF was properly assigned to its species of origin, similar isoforms were clustered together using a 99% similarity threshold. Clustering was carried out using CD-HIT 4.8.1 with options -c 0.99 -n 2 -d 0 -g 1 -sc 1 -sf 1 [86,88]. This sets the similarity threshold of the clusters to 99% and sorts the clusters by the number of sequences they contain.

Expression levels were measured for each contig by using BWA and SAMtools to map the reads from the species of origin to these final contigs. Read levels were then normalized according to length and overall number of reads using the Transcripts Per Million (TPM) formula in an attempt to correct for size bias and make the data more easily comparable to other transcriptomes [106]. A table with this information for each final toxin transcript is available in the Supplementary Materials.

The final sequences that were determined to originate from the transcriptome of *Calliophis bivirgatus* can be found in the NCBI Transcriptome Shotgun Assembly Sequence Database associated with BioProject PRJNA698476. A spreadsheet associating the codes used in our analyses and the NCBI accession numbers can be found in the Supplementary Materials.

### 3.6. Tests for Selection

Based on our phylogenetic analyses, we identified distinct clades of *C. bivirgatus* 3FTx. Coding DNA sequences for these toxins were trimmed to only include those codons which translate to the mature protein, translated, aligned, and reverse translated using AliView and the MUSCLE algorithm [107,108]. The resulting codon alignments can be found in the Supplementary Materials.

Phylogenetic trees for each clade were generated from the resulting codon alignments using the same methods as described above. These tree topologies were used for all subsequent analyses.

We used AnalyzeCodonData test in HyPhy version 2.220150316beta to estimate overall $\frac{dN}{dS}$ values [109].

### 3.7. Phylogenetic Reconstruction

Protein sequences for major toxin families (3FTx, Kunitz, and PLA_2_) were downloaded from the UniProt database [101]; protein clustering and phylogenetic analyses were used to select a subset of sequences that represent the diversity of each family. These were then combined with the translated sequences from our *C. bivirgatus* transcriptome. The sequences were aligned using a combination of manual alignment of the conserved cysteine positions and alignment using the MUltiple Sequence Comparison by Log-Expectation (MUSCLE) algorithm implemented in AliView 1.18 for the blocks of sequence in between these sites [107,108]. We reconstructed the phylogeny of these sequences using MrBayes 3.2 for 15,000,000 generations and 1,000,000 generations of burnin with lset rates=invgamma (allows rate to vary with some sites invariant and other drawn from a γ distribution) and prset aamodelpr=mixed (allows MrBayes to generate an appropriate amino acid substitution model by sampling from 10 predefined models) [110]. The runs were stopped when convergence values reached 0.01. Nexus files containing the full alignments, MrBayes settings, and output tree can be found in the Supplementary Materials. The results were visualized using Figtree v1.4.3 [111].

### 3.8. MS/MS Sample Preparation

Twenty micrograms venom proteins was dried using a lyophilizer and resuspended in 20 μL 8 M urea. To this we added 10 μL of 15 mM dithiothreitol (DTT) before incubating for 30 min at 56 ${}^{\circ }$C. Once the samples had cooled to room temperature, we added 10 μL of 15 mM iodoacetamide (IAA) and incubated for 30 min in the dark before adding another 10 μL of 15 mM DTT.

To begin enzymatic digestion, the samples had 50 μL of 50 mM ammonium bicarbonate (ABC) and 500 ng/of trypsin in dilute hydrochloric acid while sitting chilled in ice. An overnight incubation allowed the trypsin to digest the proteins in the samples. The mixture was then dried in a lyophilizer.

The dried samples were resuspended in 10 μL 2.5% acetonitrile (ACN)/1% formic acid (FA). Peptides from the samples were separated and eluted via ZipTip®(MilliPore) procedure: tips moistened with 10 μL 100% ACN repeated 3 times, tips equilibrated with 10 μL 5% ACN/0.1% FA repeated 3 times, sample loaded into tip pipetting up and down 8 times, tip washed in 10 μL 5% ACN/0.1% FA repeated 3 times, and sample is then eluted with 10 μL of 80% ACN/0.1% TFA into a new tube. The final volume of each sample was made up to 20 μL with 5% ACN/0.1% FA.

### 3.9. MS/MS

Samples were separated using reversed-phase chromatography on a Shimadzu Prominence nanoLC system. Using a flow rate of 30 μL/min, samples were desalted on an Agilent C18 trap (0.3 × 5 mm, 5 μm) for 3 min, followed by separation on a Vydac Everest C18 (300 A, 5 μm, 150 mm × 150 μm) column at a flow rate of 1 μL/min. A gradient of 10–60% buffer B over 30 min where buffer A = 1 % ACN/0.1% FA and buffer B = 80% ACN/0.1% FA was used to separate peptides. Eluted peptides were directly analysed on a TripleTof 5600 instrument (ABSciex) using a Nanospray III interface. Gas and voltage settings were adjusted as required. MS TOF scan across *m*/*z* 350–1800 was performed for 0.5 s followed by information dependent acquisition of up to 20 peptides across *m*/*z* 40–1800 (0.05 s per spectra).

Data was searched in ProteinPilot 5.0.2 using a custom database containing all contig isoforms assigned to *Calliophis bivirgatus* from our transcriptomics protocol before the clustering step, all reviewed elapid sequences from UniProt, and a database of common proteomics contaminants [101,112].

### 3.10. Visualization

Aside from specialized software mentioned in previous subsections, figures were prepared using R 3.6.2 [113], RStudio 1.1.456 [114], ggplot2 3.2.1 [115], GIMP 2.10 [116], and Inkscape 1.0 [117].

## Data Availability

All sequences used in the analysis here are available in the Supplementary Material. The sequenced reads may be found at https://www.ncbi.nlm.nih.gov/sra/SRX9989448.

## Supplementary Materials

The following are available at https://www.mdpi.com/2072-6651/13/2/124/s1, contains alignments, MSMS raw data, and transcriptome sequence information including Genbank accession codes.

## Abbreviations

The following abbreviations are used in this manuscript:

3FTx	Three-finger toxin
Na_V_	Voltage-gated sodium channel
TPM	Transcripts per million
CRiSP	Cysteine-rich secretory protein
SVMP	Snake venom metalloproteinase
MS/MS	Tandem mass spectrometry
$\frac{dN}{dS}$	Ratio of non-synonymous to synonymous substitutions
ORFs	Open reading frames
